# Functional Brain Connectivity Changes Following Vestibular Rehabilitation in Persistent Postural–Perceptual Dizziness: A Single-Case Resting-State fMRI Study

**DOI:** 10.3390/brainsci16101053

**Published:** 2026-09-30

**Authors:** Katsuhiro Adachi, Satoshi Tanaka, Naoto Sakai

**Affiliations:** 1 Sakai Neurosurgical Clinic, Tomitsuka-cho 55, Chuo-ku, Hamamatsu 432-8002, Shizuoka, Japan; 23mr01@g.seirei.ac.jp; 2 Department of Psychology, Hamamatsu University School of Medicine, Hamamatsu 431-3125, Shizuoka, Japan; tanakas@hama-med.ac.jp; 3 School of Rehabilitation Science, Seirei Christopher University, Mikatahara-cho 3453, Chuo-ku, Hamamatsu 433-8558, Shizuoka, Japan

**Keywords:** persistent postural–perceptual dizziness, vestibular rehabilitation, functional connectivity, resting-state fMRI, physiotherapy

## Abstract

Background: Persistent postural–perceptual dizziness (PPPD) is a chronic functional vestibular disorder characterized by persistent dizziness and unsteadiness. Resting-state functional magnetic resonance imaging (rs-fMRI) has identified dysfunction in vestibular, visual, somatosensory, spatial cognitive, and emotional networks in PPPD. Although vestibular rehabilitation has demonstrated efficacy in PPPD, its effects on these dysfunctional neural networks remain unclear. Case: A 37-year-old woman with PPPD had experienced recurrent rotational vertigo approximately every 6 months followed by persistent non-spinning dizziness. Assessment: Baseline assessment showed severe dizziness-related disability (Dizziness Handicap Inventory (DHI) score: 66) and depressive symptoms (Self-Rating Depression Scale (SDS): 40). Posturography demonstrated increased sway with eyes closed, whereas video head impulse testing revealed no significant abnormalities. Rs-fMRI was also performed, and functional connectivity was analyzed using SPM12 and the CONN toolbox with a region-of-interest-to-region-of-interest approach. Outcome: During the 8-week period of vestibular rehabilitation, including continued pharmacotherapy, the DHI score decreased from 66 to 16 and the SDS score from 40 to 27. Among the nine prespecified ROI-to-ROI connections, the largest longitudinal connectivity difference was observed between the right hippocampus and left frontal pole, decreasing from z = 0.110 at baseline to z = −0.277 at follow-up (Δz = −0.387). At the uncorrected exploratory level, connectivity decreased between the right anterior parahippocampal cortex and right angular gyrus but increased between the right parieto-insular vestibular cortex and right lingual gyrus. Conclusion: Clinical improvement following vestibular rehabilitation coincided with changes in PPPD-related functional connectivity, particularly reduced connectivity between the right hippocampus and left frontal pole. Although this single-patient case report had no control condition, included only one rs-fMRI scan at each time point, and involved concurrent pharmacotherapy, this finding may reflect anxiety-related mood changes in spatial cognitive processing.

## 1. Introduction

Persistent postural–perceptual dizziness (PPPD) is a chronic functional vestibular disorder [1]. The Bárány Society defined PPPD as persistent non-spinning vertigo, dizziness, and unsteadiness lasting at least 3 months [1,2,3]. Symptoms typically worsen with upright posture, active or passive motion, and exposure to complex or moving visual stimuli [3]. PPPD often develops after acute or episodic conditions that disturb balance, including peripheral vestibular disorders, migraines, anxiety, and other medical conditions [3]. Although the initial trigger may be resolved, dizziness can persist through maladaptive changes in sensory processing and postural control. Current models implicate increased visual dependence, altered postural control, and persistent anxiety or hypervigilance in PPPD pathophysiology [1,2,3]. Within this framework, anxiety and hypervigilance may influence how patients perceive and monitor motion and spatial orientation cues. Anxiety-related spatial processing is therefore a potentially relevant consideration when interpreting responses to rehabilitation.

Neuroimaging studies in patients with PPPD and related earlier diagnostic entities have provided insights into neural mechanisms relevant to persistent dizziness. In chronic subjective dizziness, a related earlier diagnostic entity, task-based fMRI demonstrated altered responses within vestibular and visual networks [4]. In patients with PPPD, resting-state fMRI has demonstrated altered functional connectivity involving vestibular, visual, sensorimotor, and prefrontal regions [5], while task-based fMRI has shown altered insular and occipital responses to simulated visual self-motion [6]. Collectively, these findings, together with previous neuroimaging reviews, suggest that PPPD involves abnormalities in sensory integration as well as attentional and emotional processing [7].

Vestibular rehabilitation has shown potential benefits in patients with PPPD. Thompson et al. reported that a habituation based vestibular and balance rehabilitation protocol improved symptoms in a pilot study, providing early support for vestibular rehabilitation as a treatment option [8]. Subsequent studies have also reported improvements in dizziness-related disability and daily functioning following vestibular rehabilitation in patients with PPPD [9,10,11]. However, the available evidence is based on studies with relatively small sample sizes and heterogeneous rehabilitation protocols, and control groups were not consistently included.

Previous neuroimaging studies have primarily examined either baseline abnormalities in patients with PPPD or acute brain responses to symptom-provoking stimulation. Baseline resting-state and task-based studies have reported abnormalities in vestibular, visual, sensorimotor, spatial cognitive, and emotional networks. In addition, Yagi et al. reported altered functional connectivity before and after visual stimulation, particularly within vestibular, visual, and spatial cognitive networks, suggesting their involvement in symptom exacerbation [12]. However, these studies examined either cross-sectional abnormalities or acute responses to experimental stimulation rather than longitudinal changes associated with treatment [5,7]. Thus, longitudinal changes in resting-state functional connectivity accompanying clinical changes during treatment remain insufficiently characterized in PPPD.

In this case report, clinical and neural changes were investigated in a patient with PPPD who received vestibular rehabilitation combined with pharmacotherapy. In addition to conventional clinical assessments, resting-state fMRI (rs-fMRI) was used to evaluate functional connectivity. The ROI-based functional connectivity analysis was guided by previously reported abnormalities in vestibular, visual, somatosensory, and spatial cognitive networks in patients with PPPD, particularly those reported by Yagi et al. [12]. The ROIs were therefore selected a priori based on previous findings; however, no specific directional hypotheses regarding longitudinal changes in functional connectivity were defined before the analysis. Accordingly, changes in functional connectivity between the two imaging time points were evaluated exploratorily. Accordingly, this case report aimed to describe longitudinal changes in clinical outcomes during an 8-week period of multimodal treatment and to explore changes in resting-state functional connectivity between two imaging time points.

## 2. Case Description (Table 1)

**Figure 1:**
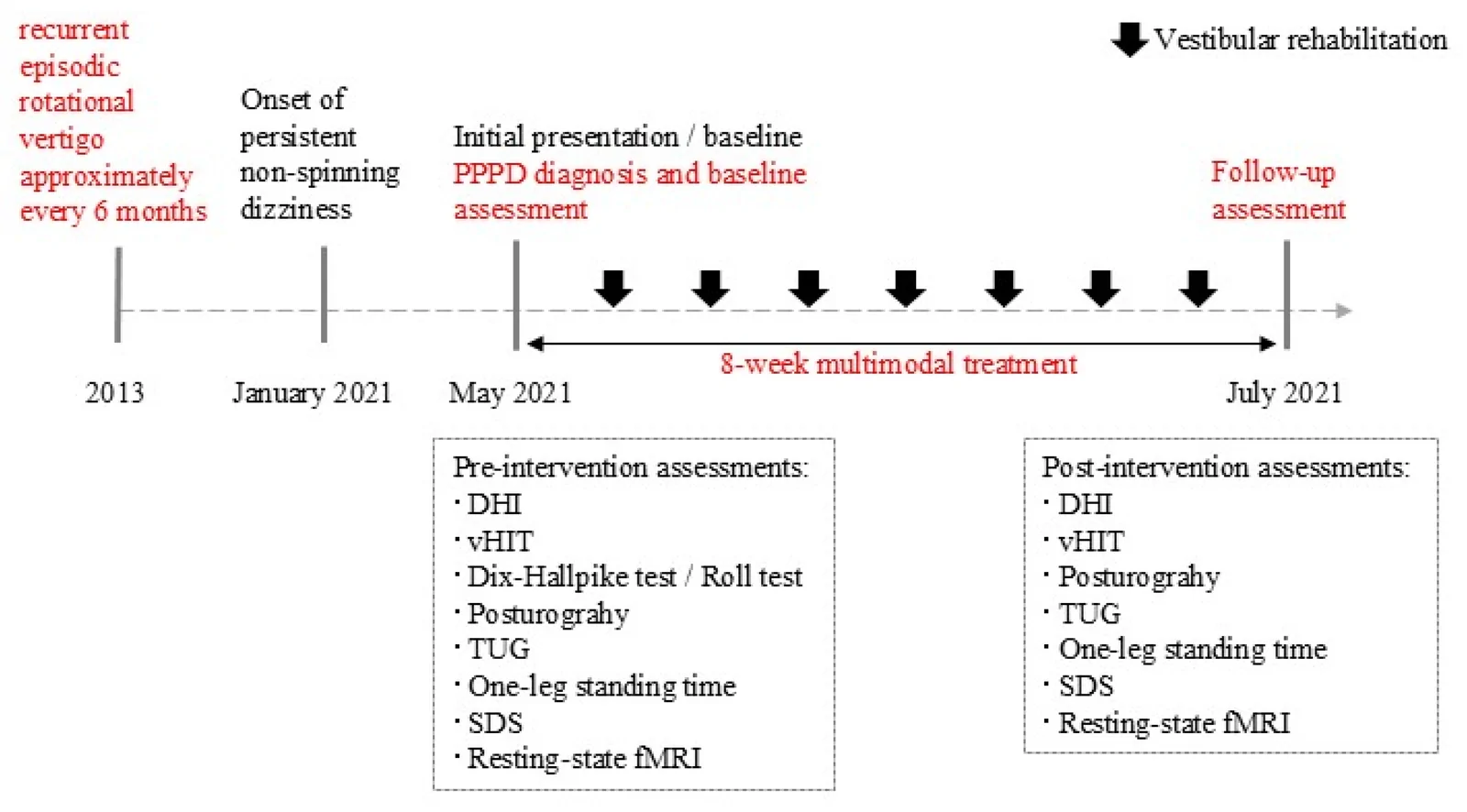
Timeline of the clinical course and intervention in a patient with persistent postural–perceptual dizziness. This timeline summarizes the patient’s clinical course from symptom onset to treatment and follow-up. The patient first experienced rotational vertigo, followed by recurrent episodes approximately once every 6 months. In January 2021, persistent non-spinning dizziness developed. In May 2021, the patient visited our clinic and was diagnosed with persistent postural–perceptual dizziness. Baseline assessments, including resting-state functional MRI, were performed at the initial visit. Vestibular rehabilitation was then administered once weekly for 8 sessions, followed by post-intervention assessments.

A 37-year-old woman presented with persistent refractory dizziness. She reported recurrent episodes of rotational vertigo approximately every 6 months for about 8 years before presentation. She also reported that she had previously been diagnosed with BPPV at another otolaryngology clinic. However, the original medical records were unavailable; therefore, the positional nystagmus findings, affected canal, treatment, and treatment response could not be verified. Accordingly, the preceding vestibular disorder was considered suspected BPPV based on the patient’s history rather than a confirmed diagnosis. She had received pharmacological treatment with betahistine mesylate and adenosine triphosphate disodium hydrate from an otolaryngologist. Four months prior to presentation, persistent non-spinning dizziness developed and was exacerbated by moving visual scenes, rapid turns, and walking through supermarket aisles. The patient had no relevant medical history, worked at a desk job, and routinely walked outdoors for 20 min each morning. Conventional brain MRI revealed no intracranial abnormalities. Based on the clinical presentation and diagnostic evaluation, PPPD was diagnosed. Baseline clinical and neuroimaging assessments were performed at the initial visit, with follow-up assessments conducted after eight weeks of treatment. The clinical course and intervention timeline are shown in Figure 1.

**Table 1:** Patient characteristics and key diagnostic findings at the initial evaluation.

Variable	
Age, years	37
Sex	Female
Diagnosis	Persistent postural–perceptual dizziness (PPPD)
History of present illness	Recurrent vertigo approximately every 6 months for 8 years before presentation; persistent non-spinning dizziness developed in January 2021.
Past medical history	None
Occupation	Desk work
Physical activity	Outdoor walking for 20 min every morning
Duration of recurrent episodic vertigo	Approximately 8 years
Duration of persistent non-spinning dizziness before presentation	Approximately 4 months
Preceding vestibular disorder	Suspected BPPV based on the patient’s history
Aggravating situations	Dizziness triggered by moving visual scenes, rapid turning movements, and walking through supermarket aisles
Brain MRI	No remarkable abnormalities
Positional tests for BPPV, right/left	
Dix–Hallpike test	Negative/negative
Roll test	Negative/negative
Video head impulse test: vHIT, VOR gain, Right/left	
Lateral semicircular canal	1.19 ± 0.07/0.99 ± 0.06
Anterior semicircular canal	1.38 ± 0.12/0.79 ± 0.05
Posterior semicircular canal	0.76 ± 0.11/1.14 ± 0.16

### 2.1. Diagnostic Assessment

The diagnosis of PPPD was made according to the Bárány Society criteria [1]. Regarding criterion A, the patient had non-spinning dizziness that persisted, with fluctuations in severity, for approximately 4 months before presentation. Regarding criterion B, the symptoms were exacerbated by upright posture and walking, rapid turning movements, and exposure to complex visual stimuli, particularly when walking through supermarket aisles. Regarding criterion C, the patient reported having previously been diagnosed with benign paroxysmal positional vertigo (BPPV) at another otolaryngology clinic, although the original diagnostic records were not available for confirmation. Regarding criterion D, the symptoms caused substantial functional impairment, as reflected by a baseline DHI score of 66, including a functional subscore of 26. The validated Japanese version of the DHI was used; the total score ranges from 0 to 100, with higher scores indicating greater dizziness-related disability, and the Japanese version has demonstrated good reliability and validity. Bilateral Dix–Hallpike and roll tests for benign paroxysmal positional vertigo were negative. Video head impulse testing (vHIT) was performed using the EyeSeeCam vHIT system (Diatec, Japan) to assess semicircular canal function. No clear low-gain pattern suggestive of peripheral vestibular hypofunction was observed. Some VOR gain values were relatively high and were therefore interpreted cautiously. Posturography was performed using a Gravicorder GW-5000 (ANIMA Corp., Tokyo, Japan). The patient was assessed once on a firm support surface under eyes-open and eyes-closed conditions, with recording durations of 60 s respectively. Posturography demonstrated increased sway with eyes closed, with sway areas of 4.05 cm^2^ and 8.76 cm^2^ with the eyes open and closed, respectively, and a Romberg ratio of 2.17. The Timed Up and Go (TUG) test was completed in 6.4 s, and one-leg standing time was 30 s on both sides. Depressive symptoms were assessed using the validated Japanese version of the Self-Rating Depression Scale (SDS), a 20-item scale with raw scores ranging from 20 to 80, with higher scores indicating greater depressive tendency. The baseline SDS score was 40, corresponding to the lower threshold for mild depressive tendency according to the Japanese raw-score interpretation. Based on the clinical history and examination findings, relevant differential diagnoses were considered. Vestibular migraine was considered less likely because there was no history or clinical features suggestive of migraine, and the patient did not report photophobia or phonophobia. Ménière disease was also considered less likely because no hearing loss was reported. Recurrent BPPV was not evident at presentation because bilateral Dix–Hallpike and roll tests were negative. The vHIT findings did not indicate clear peripheral vestibular loss, and brain MRI showed no structural abnormality that could account for the persistent symptoms. Based on the overall clinical presentation and diagnostic evaluation, no alternative vestibular or neurological disorder was considered to better account for the persistent dizziness.

Rs-fMRI was performed to assess brain networks involved in visual–vestibular integration and spatial cognition. Data were acquired using a 1.5-T scanner (SIGNA Creator; GE Healthcare, Chicago, IL, USA) with an 8-channel head coil. The rs-fMRI acquisition parameters were as follows: repetition time, 2500 ms; echo time, 40 ms; flip angle, 85°; matrix size, 64 × 64; voxel size, 3.0 × 3.0 × 3.5 mm; slice thickness, 3.5 mm; interslice gap, 0.5 mm; number of slices, 33; field of view, 19.2 cm; interleaved slice acquisition; and acquisition time, 10 min 10 s. A total of 244 functional volumes were acquired during each resting-state fMRI session, and the first four volumes were discarded before preprocessing, leaving 240 volumes for analysis. During each resting-state acquisition, the patient was instructed to keep her eyes closed and to avoid moving her head or body as much as possible. No fixation target was used. Wakefulness was not objectively monitored. The same acquisition parameters and resting-state instructions were used at baseline and at the 8-week follow-up. The phase-encoding direction could not be verified from the available imaging records. High-resolution structural images were acquired using a spoiled gradient recalled acquisition in steady state (SPGR) sequence with the following parameters: repetition time, 11.8 ms; echo time, 5.1 ms; flip angle, 15°; matrix size, 256 × 256; voxel size, 1.0 × 1.0 × 1.0 mm; slice thickness, 1.0 mm; 180 slices per slab; field of view, 25.6 cm; three-dimensional acquisition; and acquisition time, 4 min 48 s.

Functional connectivity analysis was performed using SPM12 and the CONN toolbox in MATLAB R2023b. The first four functional volumes were discarded, leaving 240 volumes for analysis. Slice-timing correction was performed for 33 slices using a repetition time of 2.5 s and an acquisition time of 2.4242 s. An interleaved ascending acquisition order was used, with odd-numbered slices acquired first and the first slice used as the reference slice.

Head-motion correction was performed using realignment, which generated six rigid-body motion parameters. The T1-weighted structural image was coregistered to the first retained EPI image. The structural and functional images were then spatially normalized to Montreal Neurological Institute (MNI) standard space, with the functional images resampled to 2 × 2 × 2 mm voxels. Spatial smoothing was performed using an 8-mm full-width at half-maximum Gaussian kernel.

Structural segmentation was performed to obtain gray matter, white matter, and cerebrospinal fluid tissue maps. Denoising was performed in the CONN toolbox. Five principal components derived from white matter and five principal components derived from cerebrospinal fluid were included as nuisance regressors. Twelve motion regressors derived from the realignment parameters were also included. Linear detrending was applied, followed by temporal band-pass filtering at 0.008–0.09 Hz after regression. Global signal was not included as a nuisance regressor. No volume censoring using scrubbing regressors was applied. Head-motion quality control was additionally assessed using framewise displacement (FD) calculated from the six realignment parameters. Rotational displacement was converted to millimeters using a 50-mm head-radius assumption. Coregistration and spatial normalization were visually inspected for both imaging sessions. The bilateral parieto-insular vestibular cortex (PIVC) ROIs were defined as 5-mm-radius spheres in Montreal Neurological Institute (MNI) space centered at x = −36, y = −25, z = 18 for the left PIVC and x = 36, y = −22, z = 17 for the right PIVC, using the same ROI definitions as Yagi et al. [12]. The remaining ROIs were anatomically defined using the FSL Harvard–Oxford Atlas included by default in the CONN toolbox. These atlas-based ROIs included the postcentral gyrus (PostCG), temporo-occipital middle temporal gyrus (toMTG), temporo-occipital inferior temporal gyrus (toITG), anterior parahippocampal cortex (aPaHC), posterior supramarginal gyrus (pSMG), angular gyrus (AG), lingual gyrus (LG), hippocampus (HC), and frontal pole (FP). Left- and right-hemispheric ROIs were treated separately in the ROI-to-ROI analysis. For each prespecified ROI pair, functional connectivity was estimated separately at baseline and follow-up and expressed as a Fisher r-to-z transformed correlation coefficient. Longitudinal change was quantified as Δz = z_follow-up − z_baseline. Because this was a single-participant, two-time-point study and the functional time series within each session were temporally autocorrelated, no inferential statistical testing of the pre–post connectivity differences was performed. The Δz values were used only as descriptive measures of the direction and magnitude of within-case connectivity changes. All nine prespecified ROI-to-ROI connections were included and reported descriptively [12].

### 2.2. Therapeutic Intervention

Vestibular rehabilitation was administered once weekly for eight sessions, with each session lasting 40 min. Pharmacotherapy with betahistine mesylate (6 mg three times daily) and adenosine triphosphate disodium hydrate (100 mg three times daily) had been initiated before the baseline assessment and was continued at unchanged doses throughout the 8-week treatment period. The exact initiation date could not be verified. In keeping with the patient’s wishes, both medications were continued without dose changes throughout the eight-week intervention period. Vestibular rehabilitation was provided once weekly for eight sessions, with each session lasting approximately 40 min. Exercise selection and intensity were individualized according to the patient’s symptoms, physical findings, and tolerance. The rehabilitation program targeted vestibular compensation, sensory integration, postural realignment, and habituation to symptom-provoking stimuli. Interventions included balance and sensory-integration training using the Redcord suspension system (Redcord AS, Arendal, Norway), postural and body-awareness training with mirror feedback, balance tasks with visual input reduced by eye closure, and exercises in which plantar somatosensory input was either enhanced or restricted. Habituation exercises involving repeated head and body movements were also performed. Task difficulty was progressively adjusted by modifying sensory conditions and postural demands when the preceding level could be performed without marked or prolonged symptom exacerbation. Supportive psychological strategies were integrated into the physiotherapy sessions but were not delivered according to a standardized cognitive behavioral therapy or psychologically informed physiotherapy protocol. To alleviate dizziness-related anxiety, the therapist provided positive feedback, addressed the patient’s concerns, and offered empathic reassurance throughout treatment. To facilitate the patient’s understanding of dizziness and the rationale for treatment, the process of integrating sensory information to maintain postural and spatial orientation was explained using an analogy of a pilot controlling an airship. This analogy was used to illustrate the role of visual, vestibular, and somatosensory information in maintaining balance and to explain the rationale for progressively challenging these sensory inputs during rehabilitation. After partial improvement in anxiety and dizziness during daily activities, graded visual exposure was introduced using videos simulating walking between supermarket aisles. The patient was instructed to perform outdoor walking for 20–30 min at least three times per week as a home program. Adherence to the home program and adverse events were not systematically recorded during the treatment period. Exact repetition counts and exercise-specific durations were not standardized or systematically recorded because the program was adjusted according to the patient’s symptoms and tolerance at each session.

### 2.3. Follow-Up and Outcomes

Follow-up assessments were conducted after the 8-week intervention. Dizziness-related disability markedly improved, with the total DHI score decreasing from 66 to 16. All DHI subscale scores also improved: physical, functional, and emotional scores decreased from 18 to 6, 26 to 6, and 22 to 4, respectively. Posturographic measures improved after the intervention. Sway area decreased from 4.05 to 3.77 cm^2^ with the eyes open and from 8.76 to 6.35 cm^2^ with the eyes closed. Sway velocity decreased from 1.57 to 1.21 cm/s with the eyes open and from 3.37 to 2.42 cm/s with the eyes closed. The Romberg ratio decreased from 2.17 to 1.68. The TUG test time showed only a small numerical change from 6.4 s to 6.15 s. One-leg standing time remained 30 s bilaterally at both assessments, with a possible ceiling effect due to the 30-s maximum testing duration. The SDS scores decreased from 40 to 27. Bilateral positional testing for benign paroxysmal positional vertigo remained negative. vHIT continued to show VOR gains within the normal range (Table 2). Mean FD was 0.219 mm at baseline and 0.139 mm at follow-up, and maximum FD was 2.891 mm and 0.968 mm, respectively. Because no scrubbing-based volume censoring was applied, all 240 retained volumes were included in the analysis at each time point. Visual inspection of coregistration and spatial normalization showed no obvious registration or normalization failure. Reliable DVARS values could not be obtained and were therefore not reported. Across the nine prespecified ROI-to-ROI connections, the largest absolute longitudinal change was observed between the right HC and left FP, decreasing from z = 0.110 at baseline to z = −0.277 at follow-up (Δz = −0.387). The baseline and follow-up Fisher r-to-z transformed correlation coefficients and their longitudinal differences (Δz) for all nine connections are presented in Table 3.

## 3. Discussion

In this patient with PPPD, vestibular rehabilitation was associated with marked improvement in dizziness-related disability after 8 weeks, with the total DHI score decreasing from 66 to 16. Improvements in the emotional and functional DHI subscales may suggest reduced dizziness-related emotional distress and fewer difficulties with daily activities, although anxiety was not directly assessed using a specific anxiety measure. In addition, the SDS score decreased from 40 to 27, indicating reduced psychological burden. Posturographic measures improved, with decreases in sway area and velocity under the eyes-closed condition and an improved Romberg ratio. However, because posturography was performed as a single trial at each assessment time point, the observed changes may partly reflect session-to-session variability and should be interpreted cautiously. Visual hypersensitivity and visual dependence are important features of PPPD [7,8]. Therefore, improved postural control with the eyes closed may indicate reduced reliance on visual input and more adaptive integration of vestibular and somatosensory information. This improvement may reflect not only symptom reduction but also recalibration of sensory integration [6,7,12]. By contrast, vHIT showed no significant abnormalities in VOR gain in any semicircular canal before or after the intervention, and one-leg standing time remained unchanged. These findings suggest that the improvement in persistent dizziness following vestibular rehabilitation may involve relearning of postural control strategies, reduced anxiety, and reorganization of brain networks involved in visual–vestibular–spatial processing [5,7]. Anxiety, hypervigilance, spatial orientation discomfort, and visuospatial performance were not directly assessed in this patient. The DHI emotional subscale and SDS cannot substitute for dedicated measures of anxiety or spatial function.

Alongside these clinical changes, the largest longitudinal connectivity difference among the nine prespecified ROI pairs was observed between the right HC and left FP, decreasing from z = 0.110 at baseline to z = −0.277 at follow-up (Δz = −0.387). This connectivity difference was interpreted as a descriptive and exploratory within-case finding rather than evidence of a statistically significant or treatment-induced neural effect. The HC is involved in spatial information processing and contextual prediction, whereas the FP contributes to higher-order attentional control and self-monitoring [5,12,13]. Behavioral studies provide additional context for considering anxiety and spatial processing in PPPD. Oertle et al. reported impaired spatial orientation after passive rotation in patients with PPPD despite preserved peripheral vestibular function; however, spatial orientation discomfort did not differ significantly between groups after adjustment for anxiety [14]. Jáuregui-Renaud et al. reported greater spatial anxiety and reduced precision of subjective visual vertical estimates in patients with PPPD. Across their combined patient and control sample, reduced precision was associated with spatial anxiety, postural sway, and depersonalization/derealization symptoms [15]. These findings support considering spatial performance and spatial anxiety alongside conventional vestibular outcomes. In this context, the observed reduction in right HC–left FP connectivity might be compatible with altered monitoring of spatial or dizziness-provoking information. However, this finding cannot establish reduced spatial anxiety or improved spatial cognition in this patient. Together with improvements in the DHI emotional subscale and SDS score, these findings suggest that vestibular rehabilitation may have altered not only the symptom severity, but also the patient’s perception and response to dizziness [12,13].

At the uncorrected level, connectivity decreased between the right aPaHC and right AG, and increased between the right PIVC and right LG. The aPaHC and AG are involved in spatial cognition and scene processing [4,8]. Reduced connectivity between these regions may indicate decreased reliance on visual–spatial processing. By contrast, the PIVC and LG contribute to vestibular and visual processing respectively, and increased connectivity may reflect more adaptive visual–vestibular integration after treatment [4,6,8,16]. The patient’s dizziness was triggered by moving visual scenes and walking between supermarket shelves. Therefore, these connectivity changes following an intervention that included graded visual exposure may be consistent with reduced visual dependence [6,8]. These connectivity differences should be interpreted as exploratory observations. Although they may be consistent with changes in multisensory or higher-order processing, the underlying mechanisms were not directly assessed in this case. Vestibular rehabilitation for PPPD included sensory-integration and balance tasks, body-awareness training, repeated head and body movements, supportive reassurance, and graded exposure to videos of supermarket aisles. These components could plausibly help the patient tolerate motion and complex visual environments, providing a possible link between rehabilitation and anxiety-related spatial processing. This interpretation is a mechanistic hypothesis, not a demonstrated effect of spatial training. Because this was a single-case, two-time-point study, conventional participant-level statistical inference of longitudinal functional connectivity differences was not possible. The reported Δz values should therefore be interpreted only as descriptive within-case changes. Head motion differed between the two imaging sessions, with higher mean and maximum FD at baseline than at follow-up. Therefore, differences in head motion may have contributed to the observed connectivity differences. Although the patient was instructed to keep her eyes closed and remain still during resting-state imaging, wakefulness was not objectively monitored. Quantitative measures of scanner stability were also unavailable. Accordingly, differences in head motion, alertness, session-to-session variability, and scanner-related variability cannot be excluded as alternative explanations for the observed connectivity differences.

Taken together, the clinical improvement may have been associated with changes in emotional responses to dizziness, visual dependence, sensory integration, and higher-order brain networks [5,7,8]. These findings suggest that vestibular rehabilitation for PPPD may promote adaptation to visual stimuli, reduce anxiety, and facilitate relearning of postural control strategies [2,4,5,8,9,10,11,17,18].

A further limitation is the absence of a PPPD-specific questionnaire. Although the DHI and SDS were administered, the Niigata PPPD Questionnaire (NPQ) was not used. The NPQ assesses symptom exacerbation associated with upright posture or walking, movement, and visual stimulation [19], which would have complemented the assessment of general dizziness-related disability. Consequently, the decrease in the DHI score cannot establish the extent of improvement in each of these PPPD-specific symptom domains. The SDS assesses depressive symptoms and likewise cannot provide this information. The available data do not allow retrospective calculation of NPQ scores.

Future studies should include a larger cohort of patients with PPPD and examine the association between clinical improvement and changes in functional brain networks using functional connectivity analysis. Such research may provide objective evidence of the effectiveness of vestibular rehabilitation on PPPD and further clarify its underlying neural mechanisms. They should also include the NPQ or another validated PPPD-specific instrument alongside general disability measures and dedicated anxiety assessments.

## 4. Conclusions

In this patient with PPPD, dizziness-related disability, psychological burden, and postural control improved during an eight-week course of vestibular rehabilitation delivered as part of multimodal care with continued pharmacotherapy. Resting-state fMRI-based functional connectivity analysis revealed descriptive differences in several brain networks, although their reliability remains uncertain given that only one scan was acquired at each time point. This case cannot establish treatment efficacy or determine which treatment component produced the improvement. Nevertheless, these observations raise the hypothesis that vestibular rehabilitation may contribute to adaptive changes in PPPD-related functional connectivity. This hypothesis warrants further investigation of vestibular rehabilitation approaches that incorporate visual adaptation, anxiety management, and postural control retraining beyond conventional vestibular compensation approaches used for peripheral vestibular disorders. Larger studies using functional connectivity analysis are needed to further evaluate the efficacy and underlying neural mechanisms of vestibular rehabilitation for PPPD.

## Figures and Tables

**Table 2:** Clinical outcomes before and after the intervention.

Outcome Measure	Pre-Intervention	Post-Intervention
Dizziness Handicap Inventory (DHI), total score	66	16
Physical subscale	18	6
Functional subscale	26	6
Emotional subscale	22	4
Video head impulse test (vHIT), VOR gain (right/left)		
Lateral semicircular canal	1.19 ± 0.07/0.99 ± 0.06	1.08 ± 0.11/1.12 ± 0.09
Anterior semicircular canal	1.38 ± 0.12/0.79 ± 0.05	1.24 ± 0.03/1.05 ± 0.05
Posterior semicircular canal	0.76 ± 0.11/1.14 ± 0.16	1.16 ± 0.07/1.12 ± 0.05
Posturography		
Sway area (eyes open/eyes closed), cm^2^	4.05/8.76	3.77/6.35
Sway velocity (eyes open/eyes closed), cm/s	1.57/3.37	1.21/2.42
Romberg ratio	2.17	1.68
Timed Up and Go test (TUG), s	6.4	6.15
One-leg standing time (right/left), s	30/30	30/30
Self-Rating Depression Scale (SDS), score	40	27

This table summarizes changes in dizziness-related disabilities before and after combined pharmacotherapy and physiotherapy. Dizziness-related disability was evaluated using the Dizziness Handicap Inventory, postural control using posturography, mobility using the Timed Up and Go test, balance using the one-leg standing test, and depressive symptoms using the Self-Rating Depression Scale.

**Table 3:** Descriptive longitudinal changes in ROI-to-ROI functional connectivity between baseline and follow-up.

ROI Pair	Pre-Treatment z	Post-Treatment z	Δz (Post − Pre)
PostCG_toMTG_R	−0.003	0.014	0.017
PostCG_toITG_R	0.013	0.016	0.003
aPaHC_R-pSMG_R	0.148	0.003	−0.144899
aPaHC_R-AG_R	0.124	−0.075	−0.199
HC_R-FP_L	0.110	−0.277	−0.387
PostCG_R-FP_L	−0.432	−0.402	0.030
PIVC_R-LG_R	−0.331	−0.103	0.228
PIVC_R-LG_L	−0.260	−0.111	0.149
PIVC_L-FP_L	−0.085	0.042	0.127

Functional connectivity values are presented as Fisher r-to-z transformed correlation coefficients. Δz was calculated as post-treatment z minus pre-treatment z. Positive Δz values indicate increased connectivity, whereas negative Δz values indicate decreased connectivity. These values are presented descriptively, and no inferential statistical testing of pre–post differences was performed. Abbreviations: PostCG, postcentral gyrus; toMTG, temporo-occipital middle temporal gyrus; toITG, temporo-occipital inferior temporal gyrus; aPaHC, anterior parahippocampal cortex; pSMG, posterior supramarginal gyrus; AG, angular gyrus; HC, hippocampus; FP, frontal pole; PIVC, parieto-insular vestibular cortex; LG, lingual gyrus; L, left; R, right.

## Data Availability

The raw data supporting the findings of this study are available from the corresponding author upon reasonable request, subject to applicable ethical and privacy restrictions.

## Abbreviations

AG	angular gyrus
aPaHC	anterior parahippocampal cortex
BPPV	benign paroxysmal positional vertigo
CONN	functional connectivity toolbox
DHI	Dizziness Handicap Inventory
rs-fMRI	resting state-functional magnetic resonance imaging
FP	frontal pole
HC	hippocampus
LG	lingual gyrus
MRI	magnetic resonance imaging
NPQ	the Niigata PPPD Questionnaire
PIVC	parieto-insular vestibular cortex
PostCG	postcentral gyrus
PPPD	persistent postural–perceptual dizziness
pSMG	posterior supramarginal gyrus
ROI	region of interest
SDS	Self-Rating Depression Scale
SPGR	spoiled gradient recalled acquisition in steady state
SPM	Statistical Parametric Mapping
TE	echo time
toITG	temporo-occipital inferior temporal gyrus
toMTG	temporo-occipital middle temporal gyrus
TR	repetition time
TUG	Timed Up and Go
vHIT	video head impulse test
VOR	vestibulo-ocular reflex
